# Suppression of cortical seizures by optic stimulation of the reticular thalamus in PV-mhChR2-YFP BAC transgenic mice

**DOI:** 10.1186/s13041-017-0320-0

**Published:** 2017-09-02

**Authors:** Wei Jen Chang, Wei Pang Chang, Bai Chuang Shyu

**Affiliations:** 1 Institute of Biomedical Sciences, Academia Sinica, Taipei, 11529 Taiwan, ROC; 2 Department of Anesthesiology and Perioperative Medicine, School of Medicine, University of Alabama, Birmingham, AL 35211 USA

**Keywords:** Channelrhodopsin, Optogenetics, Reticular thalamic nucleus, Primary somatosensory cortex, Thalamus, Seizure, GAGA antagonists

## Abstract

Deep brain stimulation in thalamic regions has been proposed as a treatment for epilepsy. The electrical current excites thalamocortical activity which is controlled by γ-aminobutyric acid (GABA)ergic interneurons in the reticular thalamic nucleus (nRT). Previous studies showed that enhancing GABAergic inhibitory strength in the nRT reduces the duration and power of seizures, indicating that the thalamus plays an important role in modulating cortical seizures. The aim of the present study was to apply optogenetics to study the role of the nRT in modulating cortical seizures. We used PV-ChR2-EYFP transgenic mice from Jackson Laboratories, in which only Channelrhodopsin-2 (ChR2) is expressed in parvalbumin-expressing interneurons. Cortical seizure-like activity was induced by electrical stimulation of the corpus callosum after applying 4-aminopyridine. ChR2 expression was abundant in the nRT and cerebellum in PV-ChR2-EYFP transgenic mice. Light stimulation in the nRT caused burst firing in regions of the thalamus and nRT in vitro. Multi-unit activity increased during high-frequency (100 and 50 Hz) light stimulation in the S1 region and thalamus in vivo. Corpus callosum stimulation-induced seizure-like activity was effectively suppressed by high-frequency (100 Hz) and long-duration (10 s) light stimulation. The suppressive effects were reversed by applying a GABA_B_ receptor antagonist but not a GABA_A_ receptor antagonist in the cortex. The results indicated that light stimulation affected thalamocortical relay neurons by activating ChR2-expression neurons in the nRT. High-frequency and long-duration light stimulation was more effective in suppressing cortical seizure-like activity. GABA_B_ receptors may participate in suppressing seizure-like activity.

## Introduction

Epilepsy is a neurological disorder that is characterized by seizure activity associated with abnormal synchronous neural overexcitation [1]. Epilepsy affects 50 million people worldwide, and nearly 0.1% die from seizure-related disorders every year in the United States. More than 30% of patients suffer from drug-resistant epilepsy [2]. For these patients, several alternative therapeutic approaches have been employed, such as transcranial direct current stimulation, transcranial magnetic stimulation, and deep brain stimulation (DBS). Deep brain stimulation has been adapted for both clinical treatment and animal studies. Different brain regions have been targeted to treat such neuronal disorders as epilepsy, Alzheimer’s disease, Parkinson’s disease, essential tremor, dystonia, cluster headache, and chronic pain [3]. Deep brain stimulation has been applied to several brain nuclei, including the cerebellum, hippocampus, amygdala, and thalamus, among others. The thalamus plays an important role in seizure development and inhibition [4, 5]. The centromedial and anterior nuclei of the thalamus have been targeted for the treatment of epilepsy. These two nuclei can suppress refractory epilepsy and generalized seizure disorder [3, 6]. Despite the successful application of DBS for the treatment of epileptic disorders, the mechanism by which the DBS acts on the nervous system is still unclear.

The reticular thalamic nucleus (nRT) is an important region that lies between thalamocortical circuits [7]. Most of the nRT contains GABAergic neurons that innervate the thalamus and produce inhibitory postsynaptic potentials (IPSPs) and provide synaptic inhibition of excitatory thalamocortical relay neurons [8]. Previous studies found that nRT stimulation can suppress limbic motor seizures [9]. Enhancing GABAergic inhibitory strength in the nRT reduced the duration and power of absence seizures [10]. Furthermore, several studies have shown that nRT plays a critical role in the modulation of cortical activity during sleep and arousal through their strong impact on thalamocortical neurons [11–14]. The aforementioned studies used electrical stimulation approaches that might lead to unwanted side effects. The nRT also receives inputs from the cortex, and thalamocortical and thalamocortical pathways lie in the vicinity of the nRT. Electrical or pharmacological stimulation of the nRT can excite these passing fibers. Optogenetic methods have recently been developed, in which light-sensitive channels are engineered to be expressed in target cells, which may provide a better method of DBS [15]. Cortical injury that was induced by thalamocortical seizures was interrupted by suppressing the activity of thalamic nuclei using a closed-loop optogenetic strategy [16]. However, the involvement of nRT in cortical seizures requires further investigation. The present study investigated whether optic stimulation of the nRT suppresses cortical seizure activity. We use transgenic mice in which Channelrhodopsin-2 (ChR2) is expressed in parvalbumin (PV)-expressing neurons in the nRT. These neurons can be specifically activated by a blue light laser (473 nm) [17]. This optogenetic approach can selectively activate the nRT and control its activity in real time, while not affecting adjacent neurons or having side effects that typically occur with traditional pharmacological or electrical stimulation methods. The present study may be critically important in laying the groundwork for understanding the role of the nRT in thalamocortical seizures.

## Methods

### Animals

Prv-mhChR2-YFP BAC transgenic mice were purchased from Jackson Laboratories (stock no. 012355, Bar Harbor, ME, USA). These mice express channelrhodopsin-2/EYFP fusion protein (mhChR2::YFP) that is directed to neuronal populations by the mouse PV promoter on the BAC transgene. Prv-mhChR2-YFP mice were genotyped using the Jackson Laboratories genotyping protocol. Polymerase chain reaction (PCR)-positive mice were mated with C57BL/6 J mice to establish transgenic lines. All of the mouse experiments were performed according to the guidelines established by the Academia Sinica Institutional Animal Care and Utilization Committee. Efforts were made to minimize animal suffering and reduce the number of animals used.

### Genotyping

To determine ChR2 gene expression in individual mice, we examined each mouse based on tail DNA. Tail samples were clipped about 0.3 cm per mice for DNA extraction, the tail samples were incubated for approximately 16 h in tail extraction buffer (50 mM Tris [pH 8.0], 100 mM ethylenediaminetetraacetic acid [pH 0.8], and 10% sodium dodecyl sulfate) that contained 20 mg/ml proteinase K (Qiagen**,** Hilden, Germany). Proteinase K was then inactivated by incubation for 5 min at 95 °C. The samples were then centrifuged at 20,000 × *g* for 1 min, and the supernatant was collected. Polymerase chain reaction amplification was used to detect the Prv-mhChR2-YFP transgene in somatic DNA. DNA polymerase was purchased from Thermo Scientific **(**Waltham, USA) and used according to the manufacturer’s instructions. The PCR conditions were the following: 95 °C for 2 min, followed by 30 × [95 °C for 30 s, 56.8 °C for 30 s, and 72 °C for 30 s], with final extension at 72 °C for 5 min. The PCR products were run on 2% agarose gels with ethidium bromide, visualized using a gel imaging system.

### Functional assessment in brain slices in vitro

Brains from 6 to 8 week old PV-ChR2 mice were sliced into 400 μm coronal sections using a vibratome (Microslicers, DTK-1000). The slices were kept fresh by artificial cerebrospinal fluid (aCSF) that contained the following: 124 mM NaCl, 25 mM NaHCO_3_, 10 mM D-glucose, 4.4 mM KCl, 1 mM NaH_2_PO_4_, 2 mM MgSO_4_, and 2 mM CaCl_2_ (pH 7.4). The slices were incubated in oxygenated aCSF (95% O_2_ and 5% CO_2_) for at least 1 h for recovery.

The recording pipettes were constructed from borosilicate glass with a pipette puller (P-97, Sutter Instruments (Novato, USA). The pipettes were filled with aCSF and inserted into the nRT or thalamic regions to record local field potentials. Blue light was generated by a blue light laser (473 nm, Sol-473-050MFL, Shanghai Dreamlaser, Shanghai, China). A 62.5 µm diameter optic fiber (GIF625, Thorlabs, New Jersey, USA) that was connected to the blue light laser was placed in the nRT region for light stimulation. Light power (422.4 mW/mm^2^) was measured by LaserCheck (LaserCheck, Coherent, California, USA) to activate expressing ChR2 neurons. Analog signals were amplified by Axon MultiClamp 700B and processed using the Power 1401 Mk II CED data acquisition system (Cambridge Electronic Design, Cambridge, England). Spike2 software (Cambridge Electronic Design, Cambridge, England) was used for offline analysis.

### Surgery for in vivo electrophysiological measurements

PV-Chr2 mice were initially anesthetized with 4% isoflurane mixed in oxygenated air and then placed in a stereotaxic apparatus. The animals were maintained under anesthesia with 1.75% isoflurane in oxygen during surgery. Body temperature was maintained at 36.5–37.5 °C with a homeothermic blanket system (Model 50–7079, Harvard Apparatus, Holliston, MA, ISA). Craniotomy was performed over the skull regions on top of the primary somatosensory cortex (S1). Small parts of the dura over S1 were carefully removed using a 23-gauge needle. After surgical preparation, the animals were anesthetized with 1.6% isoflurane during the recording session. The depth of anesthesia was periodically monitored and maintained by pinching the paw or tail so that no overt body movement occurred.

### Recording evoked field potentials in the S1 and thalamus in vivo

Extracellular field potentials that were evoked by electrical pulses were first mapped by a silver-sliver chloride (Ag-AgCl) tip electrode in the S1 region (~1.5 mm posterior and 3.5 mm lateral to bregma). To induce a cortical response, a pair of stainless-steel needles was inserted into the mouse whisker region or hind paw and used to deliver bipolar electrical stimulation (5 mA, 0.5 ms duration, model 2100, AM Systems, Washington, USA). The position in S1 that resulted in a maximal local field potential that was evoked by electrical stimulation was identified and designated as the insertion point for the multichannel recording probe (16 contact points, 100 μm interval spacing). The probe was inserted perpendicular to the S1 region. Another multichannel recording probe was used to record extracellular field potentials in the thalamus (~3.5 mm posterior and ~1.8 mm lateral to bregma; probe inserted 40° from vertical). In some of the experiments, an optoelectrode that combined an optic fiber and electrode (OAx16-10 mm-100-177, Neuronexus, Michigan, USA) was inserted into thalamic regions for simultaneous light stimulation and field potential recording. For optic stimulation, the optic fiber was inserted into the nRT for light stimulation (10–1000 ms continuous light or 10–100 Hz stimulation) by a blue light laser. An Ag-AgCl reference electrode was placed in the olfactory bulb. The sampling rate of recorded analog signals was 6 kHz, and data were processed using a multichannel data acquisition system (TDT, Florida USA) and computer.

### Cortical seizure induction

Spontaneous cortical seizure activity was induced by local application of the potassium channel blocker 4-aminopyridine (4-AP; 50 mM) on the cortical surface. A tungsten electrode was inserted into contralateral side of the corpus callosum. Electrical stimulation was performed with a 500 μA pulse current intensity, 0.5 ms pulse duration, and 10 Hz in 4 s pulse width using a constant-current pulse generator (Model 2100, A-M Systems, Washington, USA). Cortical seizure activity was induced every 10 s electrical stimulation. The induction of cortical seizure activity by electrical stimulation occurred simultaneously with optic stimulation of the nRT to evaluate possible suppressive effects.

### Chronic electroencephalographic recording and behavioral assessment in freely moving mice

A silver-ball electrode with an omnetic connector (A79014, Omnetics Connectors, Minnesota, USA) was fixed over the S1 region for electroencephalographic (EEG) recording. An optic fiber (BFL37–200, Thorlabs, New Jersey, USA) was inserted into the nRT for optic stimulation. The mice were allowed to recover from the surgical procedures at least for 1 week. EEG signals over the S1 region were recording and calculated by short-time Fourier transform (STFT). EEG signals in the S1 region were divided into shorter segments (1 s) of equal length, and the Fourier transform was computed. Computations were performed using the stft function in Matlab software (MathWorks, Natick, MA, USA). The optic fiber was inserted in the nRT and connected with a LC Ferrule (MM-FER2007C-1270, Precision Fiber Products, California, USA) and ceramic split sleeve (SM-CS, Precision Fiber Products, California, USA) that were fixed on the head by dental cement. Another optic fiber with a fiber optic rotary joint (FRJ_1X1 FC-FC, Doric Lenses, Quebec, Canada) was connected to the ferrule and sleeve to avoid optic fiber interruption when the mice were freely moving.

### Verification of electrode placement and immunohistochemistry

At the end of the experiment, a small lesion was made by passing an anodal current (25 μA, 5 s) through the deepest electrode of the electrode probe. Another lesion was made at the same lead after the electrode probe was withdrawn by 500 μm. The brains were fixed by perfusion with normal saline followed by 4% paraformaldehyde in 0.1 M phosphate-buffered saline (PBS; pH 7.4). The brains were removed, postfixed for 72 h, sectioned at 60 μm on a cryostat, and processed for immunohistochemistry. The brain slices were incubated with 1% bovine serum albumin, 0.02% sodium azide, and 0.3% Triton in PBS for 1 h. The sections were incubated with rabbit polyclonal anti-PV (GTX11427, GeneTex, California, USA) and anti-DAPI at dilutions of 1:500 for 24 h at 4 °C. Immunohistochemistry was performed using rabbit secondary IgG (H + L) antibody (pre-adsorbed, DyLight 594) and rabbit IgG (H + L) antibody (pre-adsorbed, DyLight 405, GTX 76756, GeneTex, California, USA).

### Drug administration

4-Aminopyridine (100 mM), the GABA_A_ receptor antagonist bicuculline (100 μM), and the GABA_B_ antagonist CGP52432 (100 μM) were purchased from Tocris Cookson (Ellisville, MO, USA), dissolved in physiological saline, and administered by local application on the S1 region. For seizure induction in freely moving animals, pentylenetetrazol (PTZ; 50 mg/kg), which causes alterations in excitatory and inhibitory neurotransmitter systems, was dissolved in saline and intraperitoneally injected.

### Data analysis

Field potential data were recorded by Spike2 and Signal software (Cambridge Electronic Design, Cambridge, England), and multi-unit activity in the behavioral and electrophysiological experiments was analyzed by Matlab software (MathWorks, Natick, MA, USA). Multi-unit activity was counted when the amplitude of the field response was two-times greater than the background threshold. Average EEG discharges were based on the average field potential after CC stimulation for 1 min. The EEG signals and field potentials were first averaged every 5 s (one bin) for the entire period (12 bins). The average discharges were based on the average of all 12 bins during the 1 min period. Correlations between seizure-like activity in the S1 and thalamus, recorded at different locations, were evaluated by cross-correlation analysis. Computations were performed using the xcorr function in Matlab software. If data follow normal distribution in normality test, significant changes after drug application were determined using one-way analysis of variance (ANOVA) and Systat (SPSS) software. The data were plotted using Microsoft Excel software. One-way ANOVA with repeated test was used to analyze the data of neuronal discharges in multiple groups and FFT power of EEG recordings in freely moving animals. Values of *p* < 0.05 were considered statistically significant. Tukey’s post hoc test was used to detect the sources of group differences in the ANOVA.

## Results

### Expression of channelrhodopsin

**Fig. 1:**
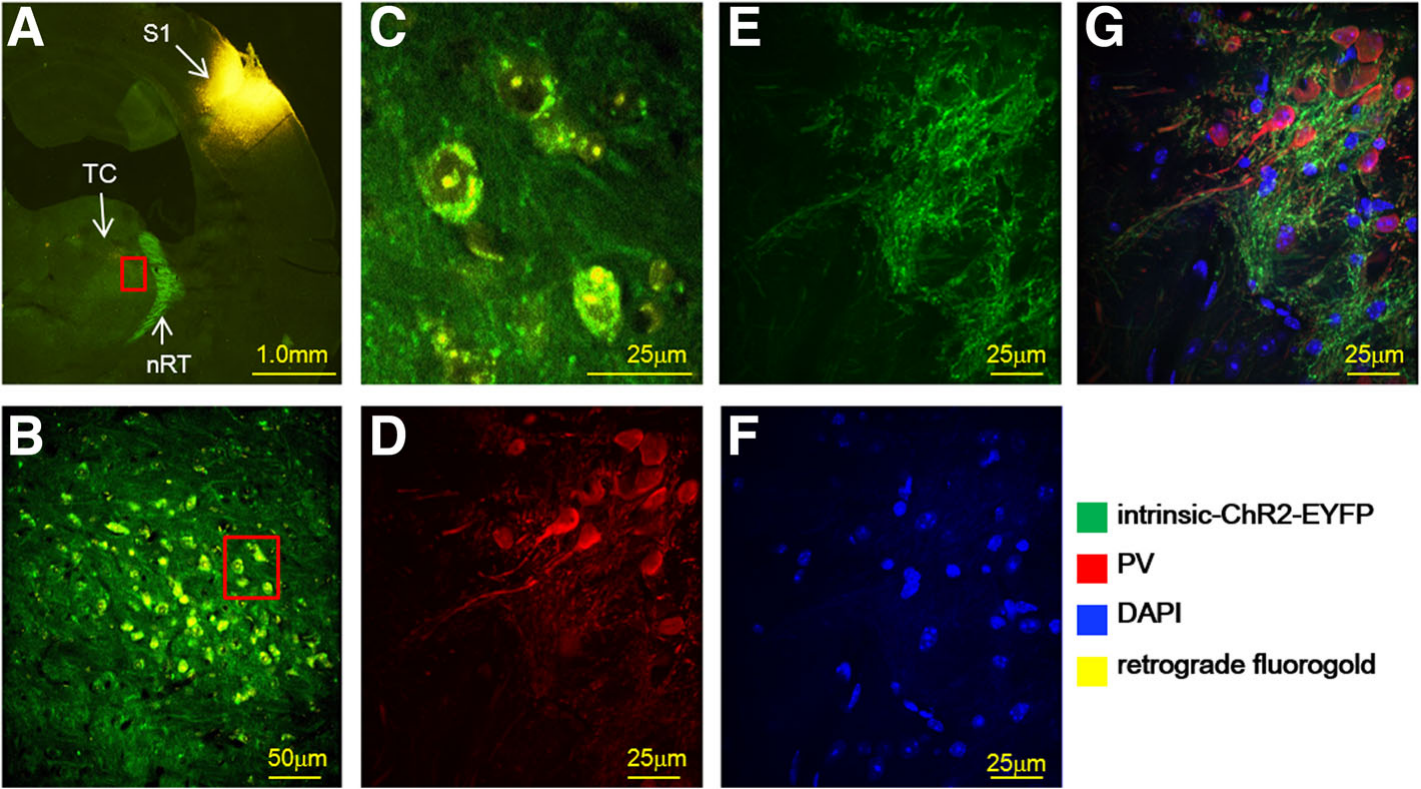
Expression of endogenous PV-ChR2-EYFP in the nRT and labeling of the retrograde dye fluorogold that was injected in the S1 region. **a** ChR2 (*green* fluorescence) and fluorogold (*yellow* fluorescence). TC denotes thalamocortical relay neurons. **b** Enlarged thalamic region that shows cells that were retrogradely labeled with fluorogold in the thalamic region. **c** Enlarged thalamic region, showing that nRT neuron fibers (*green* fluorescence) surrounded thalamocortical neurons (fluorogold retrogradely labeled neurons). **d** Immunostaining of PV in the nRT. **e** Enlarged nRT region. Endogenous ChR2 was expressed only in the nRT. **f** Chemical fluorescence stain results of DAPI in the nRT. **g** Merged image of DAPI, PV, and ChR2

ChR2 expression was found in the nRT in coronal brain sections in PV-ChR2-EYFP transgenic mice (Fig. 1a). Green fluorescence indicates endogenous ChR2 expression. The retrograde dye fluorogold was injected into the S1 region to confirm the thalamocortical pathway (Fig. 1a). Retrogradely labeled fluorogold dye was detected in thalamic neuron fibers (yellow fluorescence) and nRT neuron fibers (green fluorescence), which innervated thalamic neurons (Fig. 1a [square enlarged in Fig. 1b], Fig. 1b [square enlarged in Fig. 1c]). Most neurons in the nRT were PV-type interneurons (Fig. 1d, red fluorescence). Endogenous ChR2 was expressed on PV interneuron membranes in the nRT region (Fig. 1e, g, green fluoresce). Parvalbumin interneurons and ChR2 and DAPI staining are merged in Fig. 1g.

### Functional verification of ChR2

**Fig. 2:**
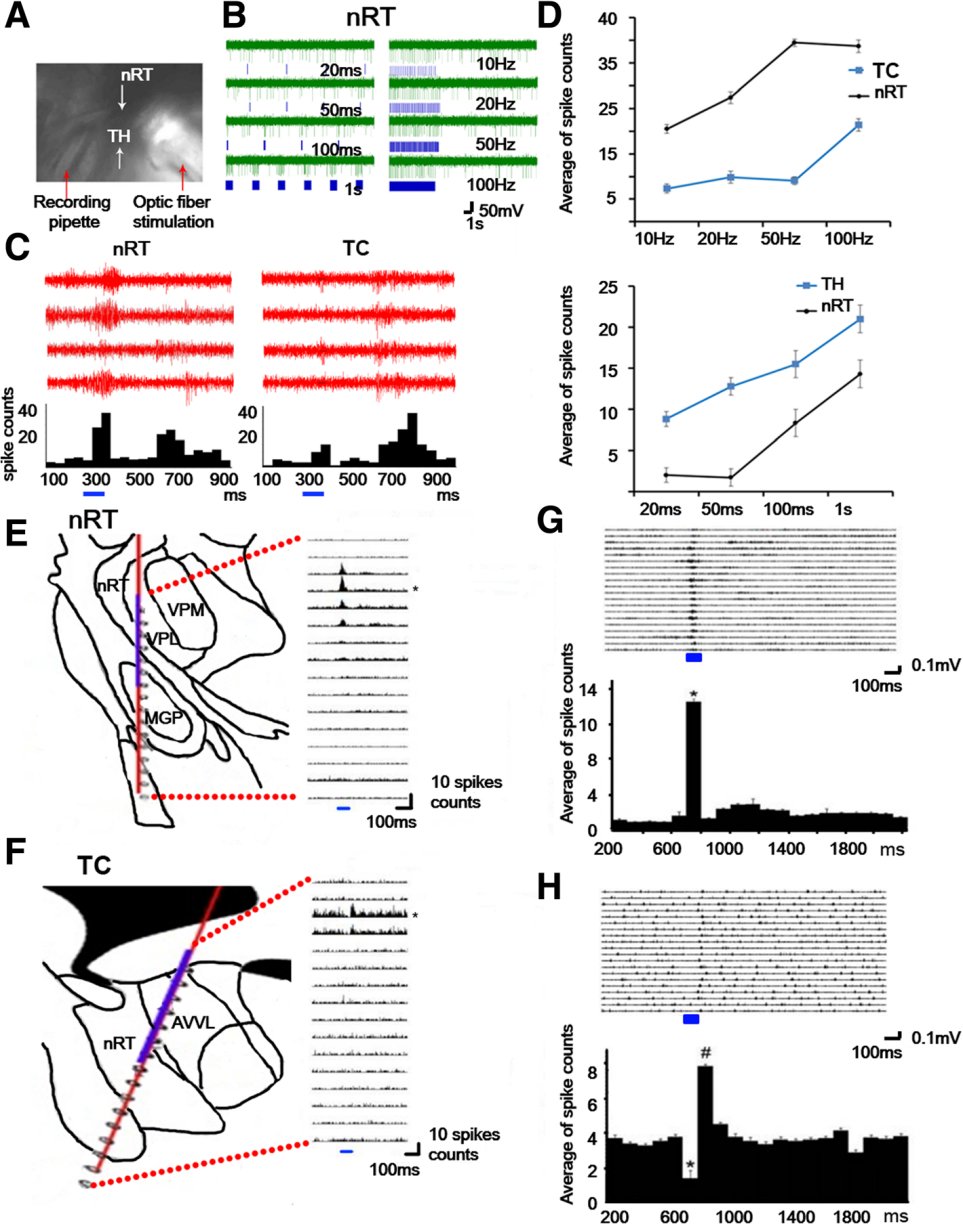
Functional verification of ChR2 in vitro (**a-d**) and in vivo (E-H)*.* **a** Horizontal brain slice (400 μm) that contained the nRT and thalamus. **b** nRT neurons in response to 10 Hz, 20 Hz, 50 Hz, 100 Hz, 20 ms, 50 ms, 100 ms, and 1 s light stimulation. **c** Spikes from nRT (*left*) and thalamocortical relay neurons (TC) (*right*) neurons in response to light stimulation. Multi-unit activity in the nRT significantly increased during light stimulation. Multi-unit activity in the thalamus was slightly enhanced during light stimulation and significantly increased 500 ms after light stimulation (*blue*). **d** Averaged spike counts from nRT and thalamus neurons in response to different pulse durations of light stimulation. **e** Position of optoelectrode aligned on the histology atlas. Upper channels 3–7 are in the nRT. (Right) Summation of multi-unit activity from 10 sweeps. During laser stimulation (100 ms blue line), spike activity was evoked in channels that were located in the nRT. **f** Position of optoelectrode aligned on the histology atlas. Upper channels 2–9 are in the thalamus. (Right) Summation of multi-unit activity from 10 sweeps. During laser stimulation (*blue* line), spike activity decreased and rebounded in channels that were located in the thalamus. **g** Multi-unit activity from 20 sweeps in channel 4 in the nRT. **h** Multi-unit activity from 20 sweeps in channel 3 in the thalamus. During laser stimulation, multi-unit activity in the thalamus significantly decreased and rebounded after laser stimulation was terminated

Brain slices that contained the nRT and thalamus were prepared to verify the functional activation of ChR2. A blue light laser (473 nm) was used to stimulate the nRT, and multi-unit activity was recorded in the nRT and thalamus (Fig. 2a). Multi-unit activity was induced in the nRT by different light frequencies and durations (Fig. 2b). Burst firing with short and long latencies was optically stimulated in the nRT and recorded in the nRT and thalamus (Fig. 2c). Spike counts that were evoked by different light stimulation conditions are shown in Fig. 2d. To test the functional activation of ChR2-expressing neurons in vivo, an optoelectrode was inserted in the nRT (Fig. 2e) and thalamus (Fig. 2f) to record multi-unit spike responses to light stimulation. Twenty trials of optic stimulation in the nRT (Fig. 2g) and thalamus (Fig. 2h) were averaged. A rebound response was detected after light stimulation in the thalamus (Fig. 2h, bottom). These results indicate that ChR2-EYFP neurons were activated by blue light stimulation and relayed signals to lateral thalamic regions.

### Cortical and thalamic responses evoked by different light stimulation durations and frequencies

**Fig. 3:**
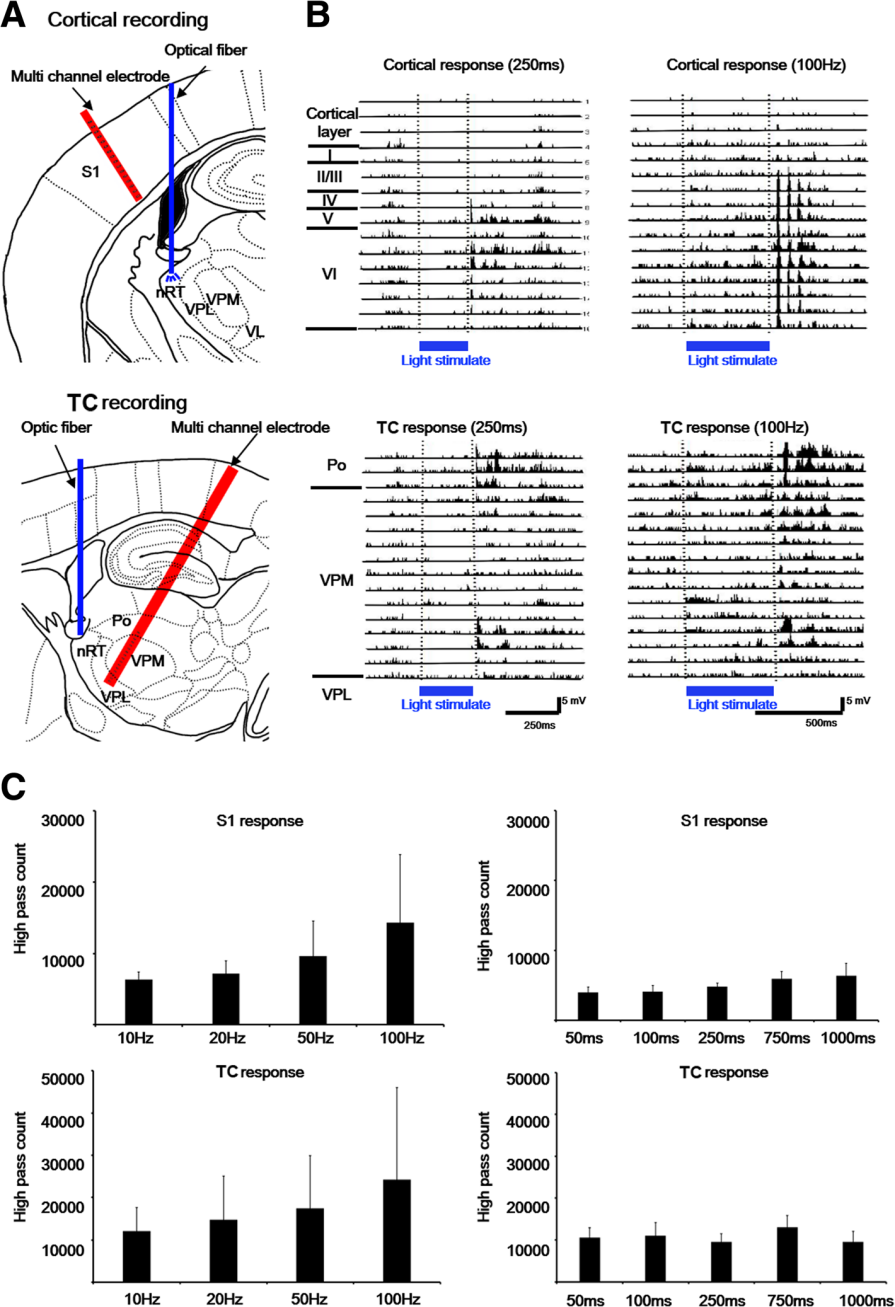
Cortical and thalamic responses with different durations and frequencies of light stimulation. **a** In the upper panel, the multichannel electrode is positioned in S1. One optic fiber was implanted in the nRT. In the lower panel, another multichannel electrode was located in the VPM. **b** High-pass field potentials of S1 and thalamus after different light stimulation conditions in the nRT. **c** Statistical analysis of spike counts recorded in the cortex and thalamus with different light stimulation conditions in the nRT, the target brain area S1 showed that non-significant differences occurred at different durations of stimulation (F4, 20 = 1.70, *p* > 0.05) and frequency of stimulation (F3, 15 = 0.82, *p* > 0.05) after one-way ANOVA with repeated test. In addition, another target side thalamus showed that non-significant differences occurred at different durations of stimulation (F4, 20 = 0.59, *p* > 0.05) and frequency of stimulation (F3, 15 = 1.37, *p* > 0.05, *n* = 6)

To test thalamic and cortical responses to light stimulation in the nRT, multichannel recording electrodes were inserted in the S1 and thalamus (Fig. 3a). Optic light stimulation-evoked multi-unit activity was recorded and analyzed in the S1 region and thalamus (Fig. 3b). Firing activity in thalamocortical relay regions (S1 and thalamus) responded to different durations and frequencies of light stimulation in vivo. Multi-unit activity increased during high-frequency (100 and 50 Hz) light stimulation in the S1 region and thalamus (Fig. 3c). These results indicate that optic light stimulation in the nRT induced multi-unit activity through the thalamocortical pathway to the S1 region.

### Closed loop of cortical and thalamic seizure activity

**Fig. 4:**
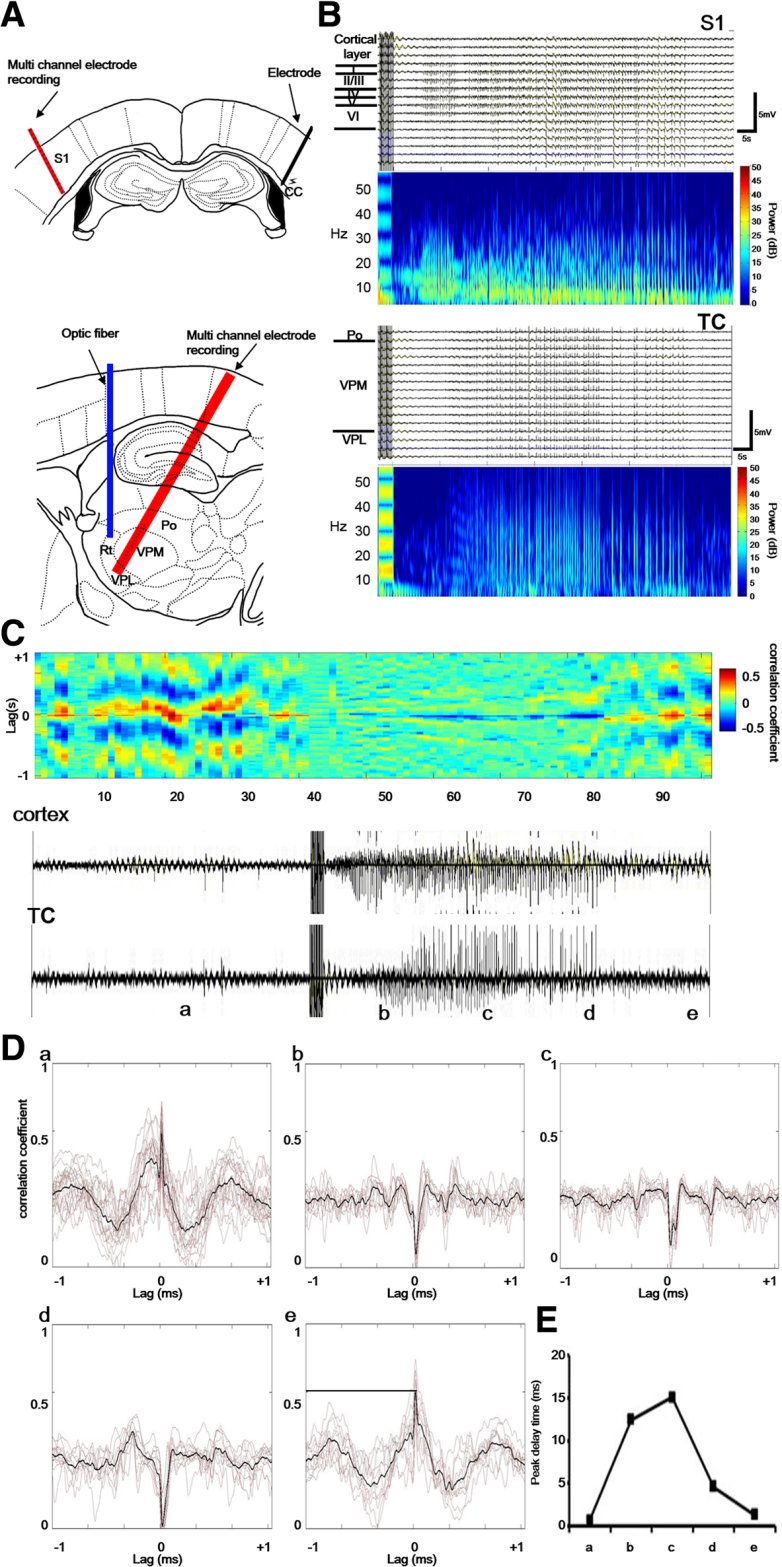
Closed-loop cortical and thalamic seizure activity was induced by corpus callosum stimulation after applying 4-AP. **a** The left panel shows the electrical probe location in the corpus callosum (*blue bar*) and a multichannel recording probe in the S1 and thalamus. The optic fiber was inserted above the nRT. **b** The right panel shows field potentials that were recorded by a multichannel electrode in S1 and the fast Fourier transform (FFT). The FFT power significantly increased at the onset of seizure activity. Vertical lines are artifacts from corpus callosum stimulation. Seizure oscillations in the S1 and thalamus were recorded after corpus callosum stimulation. **c** Cross correlation of cortical and thalamic seizure activity. The sweep period was divided into pre-seizure (**a**) and post-seizure (**b**-**d**) periods. **d** Cross correlograms of activity in periods **a** to **e**. The results showed a positive correlation in the pre-seizure period (**a**) and negative correlations in the post-seizure periods (**b**-**e**). **e** Plot of peak latency of cross correlation coefficients during the post-seizure periods (**b**-**e**)

Cortical seizures were induced by the local application of 4-AP (100 mM) on the S1 surface and electrical stimulation of the contralateral corpus callosum (Fig. 4a). Seizure-like activity was recorded by simultaneously detecting local field potentials and frequency spectra in the S1 region and thalamus (Fig. 4b). The sweeps of seizure activity were divided into five time bins. Cross correlations of seizure-like activity (Fig. 4c, lower panel) were analyzed and are presented as correlograms (Fig. 4c, upper panel). Correlation coefficients of the relative bins between S1 and the thalamus indicated positive correlations (*r* = 0.5904) in the pre-seizure period (Fig. 4 Da). Correlation coefficients between S1 and the thalamus became negative (*r* = −0.4008 [Fig. 4 Db], *r* = −0.3348 [Fig. 4Dc], *r* = −0.4884 [Fig. 4Dd], *r* = 0.4992 [Fig. 4De]) during the electrically induced seizure-like activity period (Fig. 4Cb-d). During the seizure onset period, the peak time of the cross-correlation coefficient was delayed, with a maximal peak latency of 15 ms (Fig. 4e). These results show that seizures were initiated in the cortex and then propagated to the thalamus, indicating that seizure-like activity in the cortex and thalamus was highly correlated. Seizure-like activity in the cortex preceded thalamic activity and was initiated earlier. Thus, thalamic activity was affected by seizure progression though the thalamocortical pathway.

### Suppressive effects of the nRT on electrically evoked cortical seizures

**Fig. 5:**
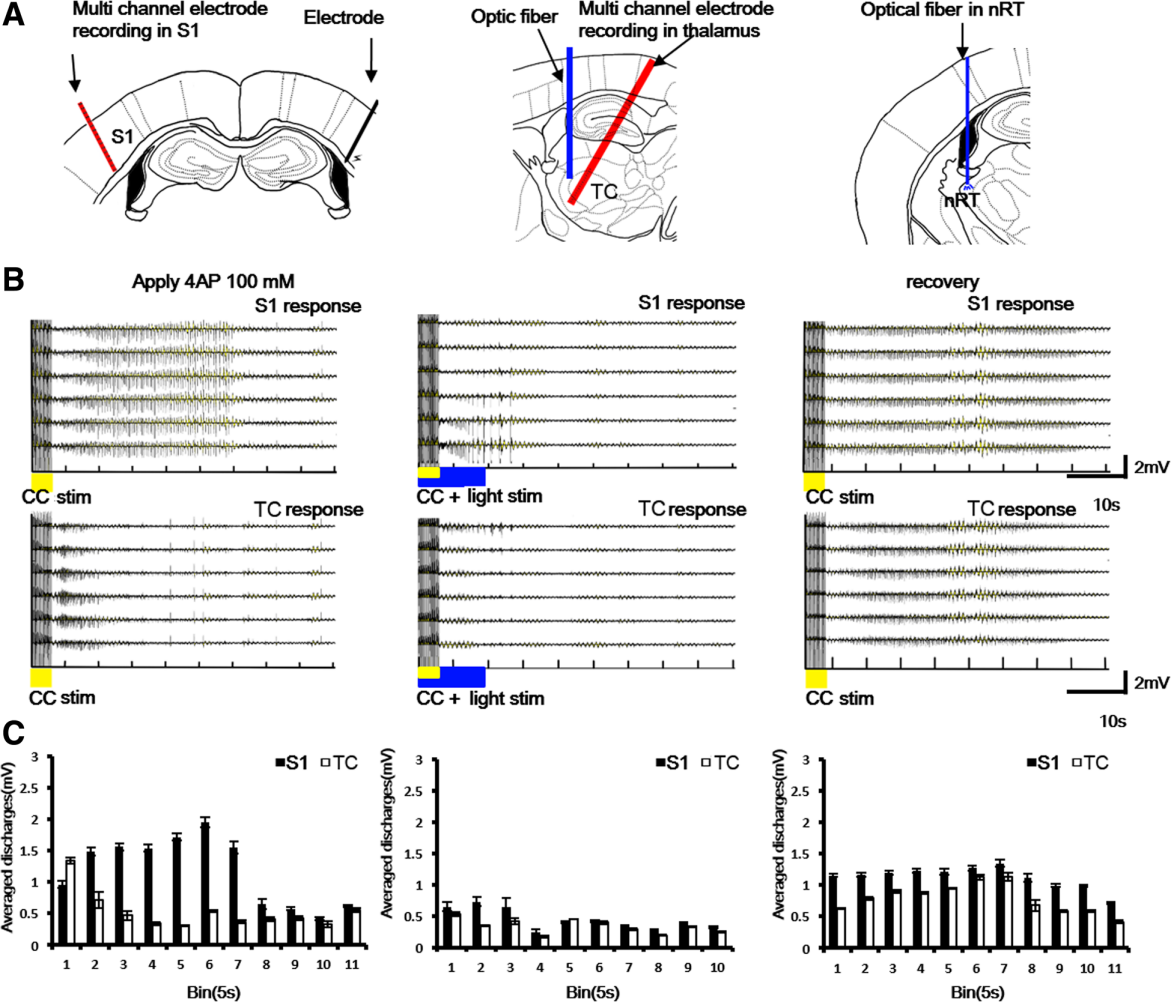
Example of cortical and thalamic seizure activity that was suppressed by nRT stimulation. **a** Probe and stimulation sites. Optical fiber targeted on the nRT. Multichannel recording electrodes were placed in S1 and VPM&VPL of the thalamus. **b** In the *left* panel, cortical and thalamic seizure-like activity was induced by electrical stimulation of the corpus callosum (4 s, 10 Hz, 500 μA) (*yellow bar*) after applying 4-AP. In the middle panel, light stimulation (*blue bar*) was applied immediately following electrical stimulation. In the right panel, only electrical stimulation was applied without light stimulation. **c** Effect of optic stimulation on seizure activity. In the middle panel, sweeps were analyzed every 5 s. Corpus callosum stimulation-induced seizure-like activity was suppressed by 10 s light stimulation (*blue mark*) after corpus callosum stimulation. In the *right* panel, seizure activity recovered when light stimulation in the nRT was terminated

**Fig. 6:**
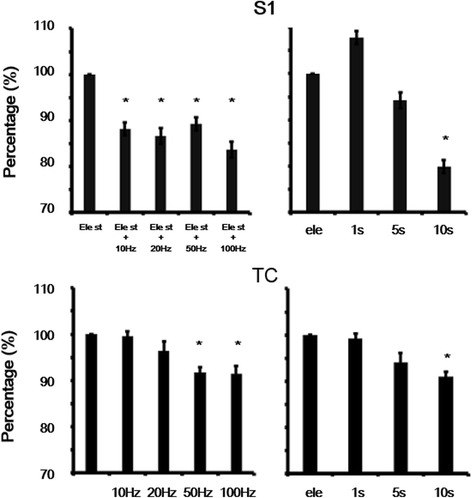
Statistical analysis of suppressive effect of light stimulation on cortical and thalamic seizures. Various frequencies (10 Hz ~100 Hz, 10 s duration) and durations (1 s ~ 10s) of continuous light stimulation were applied. High-frequency 10s and long-duration continuous light stimulation significantly decreased seizure activity. *p < 0.05, compared with electrical stimulation only (one-way ANOVA with repeated test followed by post hoc test). Error bars indicate the SEM

The setup for cortical seizure induction, recording, and optic stimulation is shown in Fig. 5a. Spontaneous cortical and thalamic seizure-like activity was induced by the application of 4-AP and could be induced by electrical stimulation of the contralateral corpus callosum (Fig. 5b, left panel, yellow). This seizure-like activity was significantly suppressed by 10 s light stimulation and recovered after the light stimulation was terminated (Fig. 5b, middle and right panels). Sweeps of seizure activity were analyzed every 5 s. Corpus callosum activation-induced seizure-like activity was suppressed by 10 s light stimulation after corpus callosum stimulation. Seizure activity recovered when light stimulation of the nRT was terminated (Fig. 5c). The suppressive effect was tested using various frequencies (10 Hz ~100 Hz, 10 s duration) and durations (1 s ~ 10s) of continuous light stimulation. Corpus callosum activation-induced seizure-like activity was effectively suppressed by high-frequency (100 Hz) and long-duration (10 s) light stimulation. The one-way ANOVA with repeated test (electrical stimulation vs. different light stimulation conditions) indicated significant effects of group in the S1 region (*F*_7,564_ = 19.61, *p* < 0.05) and thalamus (*F*_7,385_ = 4.9, *p* < 0.05). The suppressive effects were significant for both high-frequency and long-duration light stimulation (Fig. 6). The distribution of lesion sites indicated that multichannel probe recording sites were located in the thalamus and nRT. These results demonstrate that corpus callosum activation-induced cortical seizures could be effectively suppressed by light stimulation of the nRT.

### Effects of GABA antagonism on cortical seizure suppression

**Fig. 7:**
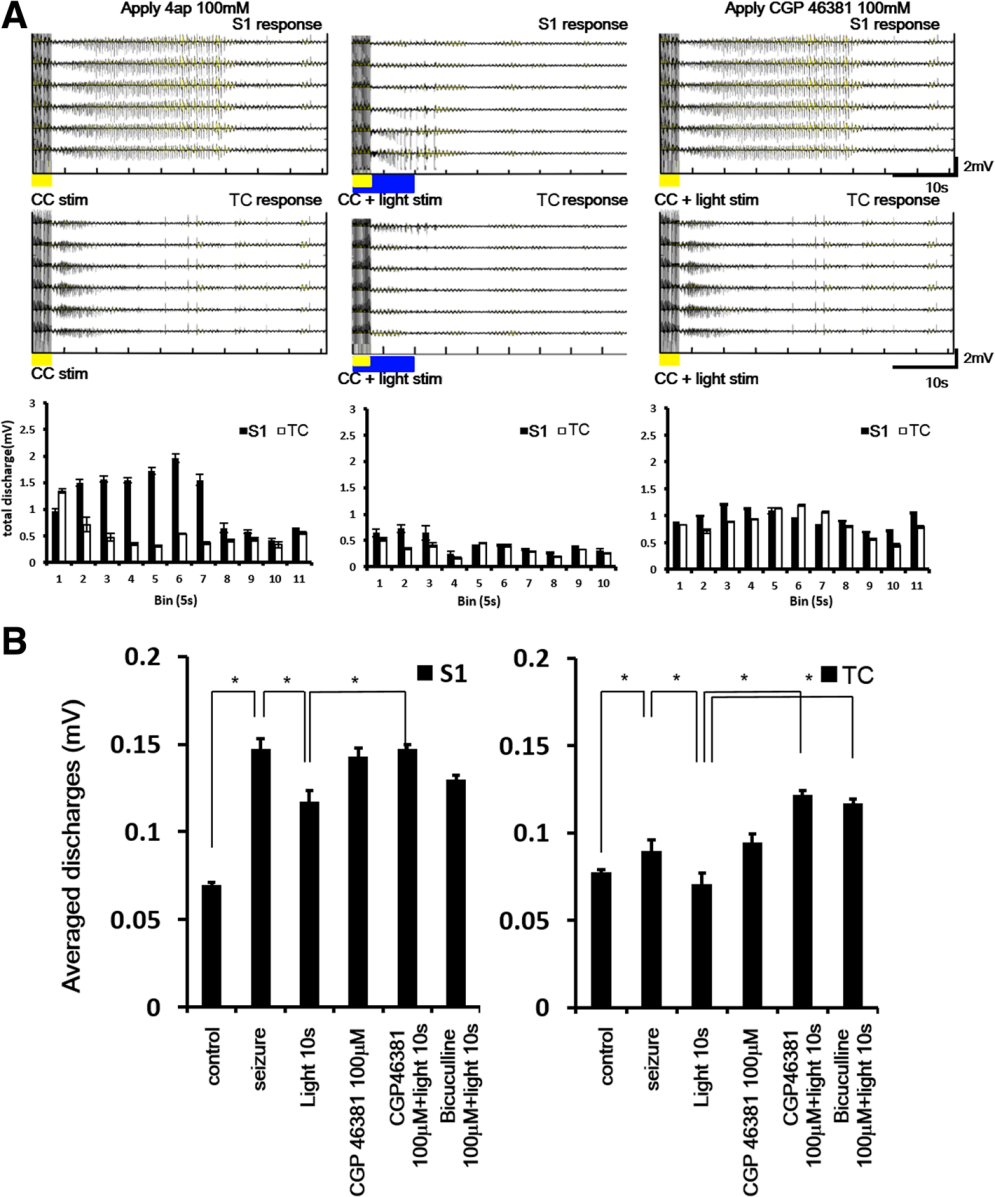
Seizure-like activity was partially reversed by the GABA_B_ antagonist CGP46381 (100 μM). **a** In the left panel, cortical and thalamic seizure-like activity was induced by electrical stimulation of the corpus callosum (yellow bar) after applying 4-AP. In the middle panel, corpus callosum stimulation-induced seizure-like activity was suppressed by 10 s light stimulation (*blue mark*) after corpus callosum stimulation. In the right panel, seizure-like activity was partially reversed by the GABA_B_ antagonist CGP46381 (100 μM). **b** Average discharges induced by different light stimulation conditions and drug applications in the S1 region (*left panel*) and thalamus (*right panel*). The suppressive effects of 10 s light stimulation were reversed by the GABA_B_ antagonist. *p* < 0.05, one-way ANOVA with repeated test followed by post hoc test. *n* = 3

The suppressive effect may be caused by feedforward inhibition that is mediated by GABA receptors [18, 19]. The effects of GABA_A_ and GABA_B_ antagonism were tested by applying receptor antagonists on the cortical surface. Seizure-like activity in the S1 region and thalamus was induced by applying 4-AP (100 mM) and electrical stimulation of the corpus callosum (Fig. 7a, left). Seizure-like activity was suppressed by 10 s light stimulation (Fig. 7a, middle). Application of the GABA_B_ antagonist reversed these suppressive effects (Fig. 7a, right). Figure 7b shows the effects of application of the GABA_A_ and GABA_B_ antagonists on the suppressive effects, summarized as average cortical evoked discharges. Statistical analysis showed that averaged discharges were significantly different in different treatments. Seizure activity was significantly decreased by light stimulation of the nRT for 10 s. These suppressive effects were abolished by application of the GABA_B_ receptor antagonist. The GABA_A_ antagonist bicuculline was less effective than the GABA_B_ antagonist CGP52432. The one-way ANOVA with repeated test (electrical stimulation vs. different light stimulation conditions) indicated significant effects of group (*F*_5,439_ = 51.30, *p* < 0.05). The suppressive effects of light stimulation could be reversed by applying the GABA_B_ antagonist but not the GABA_A_ antagonist (Fig. 7b, left panel). Seizure activity in the thalamic region was also suppressed by 10 s light stimulation, and this suppressive effect was reversed by the GABA_B_ antagonist and GABA_A_ antagonist (*F*_5,479_ = 165.444, *p* < 0.05; Fig. 7b, right panel).

### Evaluation of optic stimulation of the nRT in awake freely moving mice

**Fig. 8:**
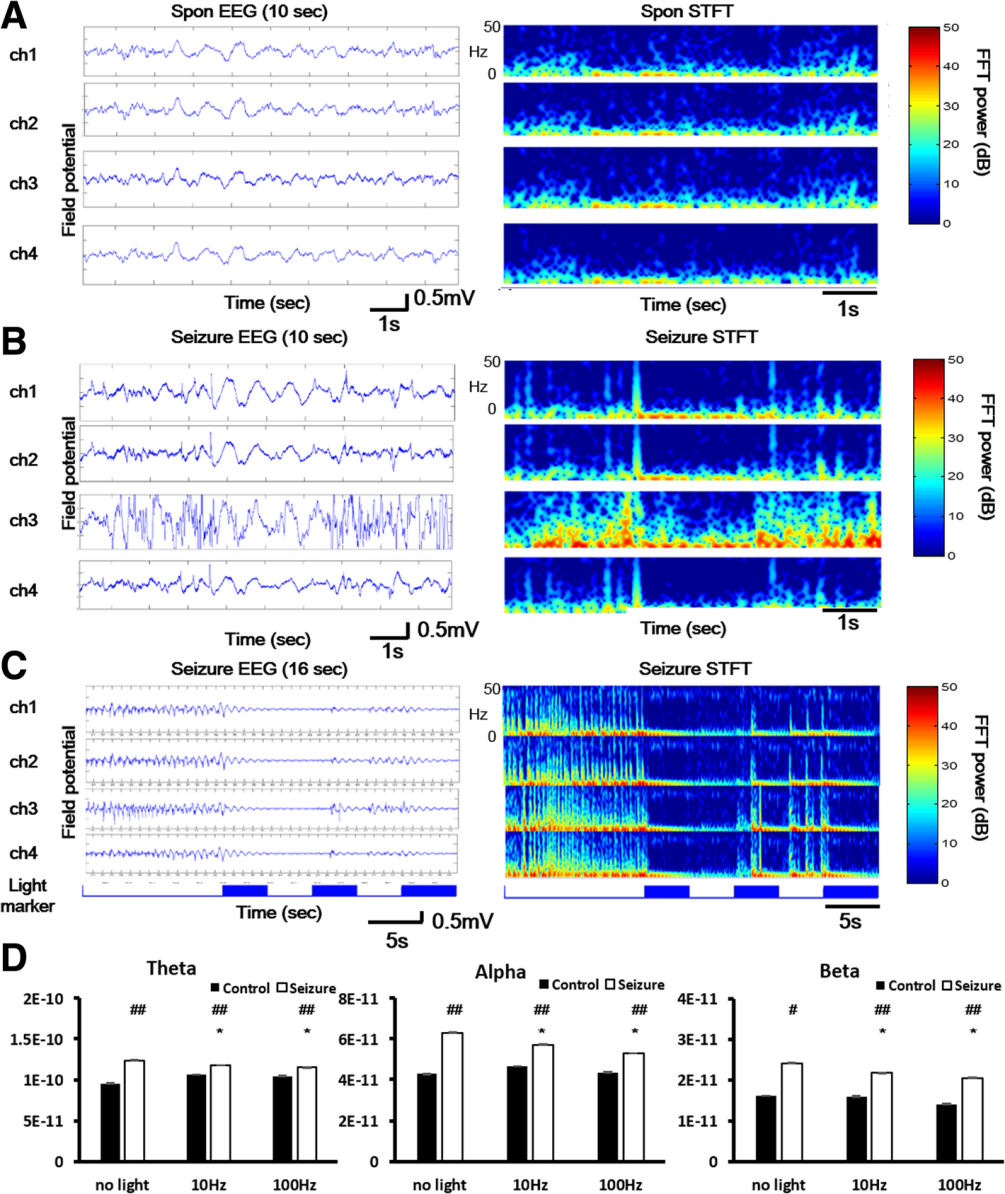
Seizure-like activity was suppressed by light stimulation in the nRT in freely moving mice. **a** The left column shows spontaneous field potentials in the S1 region based on EEG recordings. The right column shows spontaneous field potentials after STFT. **b** Seizure-like activity was induced by PTZ (50 mg/kg, i.p.). The right column shows the field potentials of seizure-like activity. **c** The left column showed EEG waves in the S1 region in seizure onset period. Blue marks represent the light stimulation periods in the nRT. Seizure like activities were suppressed when light stimulation turned on. Right column presented STFT in seizure-like activities and seizure suppression period. **d** Different light stimulation conditions in the pre- and post-seizure periods. FFT power was analyzed and divided into three frequency bands (theta, 4–7 Hz; alpha, 8–15 Hz; beta, 16–30 Hz). In the post-seizure periods, the frequency power significantly decreased in all three bands when 10 and 100 Hz light stimulation was applied. *p < 0.05 compared with different light stimulation, ^##^ *p* < 0.05 compared with control and seizure groups. Error bars indicate SEM. (one-way ANOVA with repeated test followed by post hoc test)

Silver-ball electrodes with an omnetic connector were attached to the cortical surface over S1 to record EEG activity in freely moving animals. Spontaneous local field potentials (Fig. 8a, left panel) and STFT (Fig. 8a, right panel) were recorded in freely moving animals. The theta band frequency was noted in freely moving animals. Electroencephalographic recordings showed that seizure activity spikes were induced 5 min after the intraperitoneal injection of PTZ (Fig. 8b). Light stimulation (100 Hz, 5 s) was delivered during the period of seizure induction. Seizure activity spikes were suppressed by light stimulation of the nRT (Fig. 8c, blue). Different light stimulation conditions (control without light stimulation, 10 Hz light stimulation, 100 Hz light stimulation) were applied during the pre-seizure period (black column) and seizure period (white column) in the S1 region. The statistical analysis of different frequency wave bands showed that the power of all of the wave bands was significantly decreased by low-frequency (10 Hz) and high-frequency (100 Hz) light stimulation compared with the seizure period without light stimulation. The one-way ANOVA with repeated test revealed significant effects for each band (theta band, *F*_2,496_ = 151.572; alpha band, *F*_2,2399_ = 403.663; beta band, *F*_2,5999_ = 647.974; all *p* < 0.05). These results indicated that GABAergic neurons in the nRT played an important role in suppressing cortical seizure-like activity in freely moving animals.

## Discussion

The present study found that 4-AP-induced seizures in the cortex and thalamus were suppressed by optogenetically activating the nRT. We also found that the suppressive effect of light stimulation on cortical seizures was related to GABA_B_ receptor transmission. Overall, cortical seizures were effectively suppressed by light stimulation in freely moving mice.

ChR2-EYFP was abundantly expressed in PV-expressing neurons in the nRT in Prv-mhChR2-YFP BAC transgenic mice, and these neurons responded to light stimulation. ChrR2 has been shown to have low expression in the thalamus, striatum, cortex, and hippocampus [17, 20]. The low transgene expression in the cortex may be caused by transcription regulatory elements that are inherent to the BAC construct or post-translational processes. Low expression can be rescued by reconstructing PV-expressing neurons with tdTomato, and these neurons were shown to be expressed throughout the nRT and other areas [21]. We further found innervation of thalamocortical projection neurons by these nRT neurons, confirmed by the observation of connections between extended processes of PV-ChR2 neurons and the retrogradely labeled thalamic neurons. The nRT has two types of interneurons: projection neurons that innervate thalamic relay neurons and local interneurons. ChrR2 is expressed in both types of neurons, and the optical stimulation likely indiscriminately excited both types of neurons. The effects of excitation would be conveyed by projection neurons and exert effects on thalamic relay neurons. The functional role of GABAergic interneurons in the nRT was demonstrated in a recent study in which deletion of the GABA_A_ receptor α1 subunit increased inhibition in the nRT and reduced absence seizures, in which abnormal oscillations are believed to develop in thalamocortical pathways [22–24].

4-Aminopyridine has been widely used to induce focal epileptic discharges in different region in vitro, including the hippocampus [25], cingulate cortex [26–28], neocortex [29], amygdala [30], brain slice of guinea pig [31] and optical imaging study in Ferrets in vivo [32]. The application of 4-AP in the somatosensory cortex induced seizure-like activity and affected the neighboring cortex [33]. Our previous studies showed that the thalamus regulates 4-AP-induced cingulate cortex seizure-like activity by regulating gap junctions [28, 34]. Seizure-like activity that was induced by 4-AP application in the somatosensory cortex may have affected ventrobasal complex (VB) regions through the thalamocortical pathway, revealed by synchronous discharges in the cortex and thalamus (Fig. 4). Cortical stroke-induced seizure-like activity is known to produce long-distance synchronization in the cortex and thalamus [15]. Long-distance synchronization is modulated by ascending cholinergic and serotonergic systems and thalamocortical feedback loops. When seizures occur, extracellular K^+^ concentrations increase and extracellular Ca^2+^ concentrations decrease, leading to changes in synaptic transmission, in which low Ca^2+^ concentrations inhibit the normal release of transmitters [34]. 4-Aminopyridine induced seizure-like activity in the cortex, which may have altered synaptic neurotransmitter release and led to synchronous oscillatory activity through thalamocortical feedback loops.

Electrical stimulation of the nRT has been suggested for the treatment of temporal lobe epilepsy [10]. However, electrical stimulation has side effects. Electrical stimulation can affect neurons that surround the target region, and all types of neurons are activated by such stimulation. Furthermore, damage or lesions can occur in the target region if the stimulation duration is too long. Optogenetics may solve these problems by activating specific neuronal groups. Previous studies used this new optogenetic method to control thalamic activity to suppress cortical injury-induced seizures [16] and to control different types of interneurons for the treatment of epilepsy [35]. In the present study, we used different durations and frequencies of light stimulation of the nRT. Seizure activity and behavior were suppressed by nRT neuron activation (Figs. 5 and 8). These suppressive effects were more robust with high-frequency and long-duration light stimulation, which may have resulted from augmented GABA release when the nRT was activated. Previous studies found that a halorodopsin-expressing adeno-associated virus in the hippocampus suppressed kainic acid-induced temporal lobe epilepsy [36, 37]. Neocortical epilepsy is usually drug-resistant. A halorodopsin-expressing lentivirus also suppressed tetrodotoxin-induced focal cortical seizures [38]. The inhibition of neuronal activity in the CA3 area of the hippocampus suppressed kainic acid-induced seizures [39, 40]. Seizures that were induced by cortical stroke through the thalamocortical pathway were suppressed by the halorodopsin-induced inhibition of VB neuron activity [16]. High-frequency optic stimulation (20 and 50 Hz) of the CA3 area of the hippocampus suppressed 4-AP-induced seizures in Thy1-ChR2 transgenic mice [41]. We also observed suppressive effects of high-frequency optic stimulation. Such high frequencies may thus be better for regulating GABA neurotransmitter release in the nRT to suppress cortical seizures.

The suppression of cortical seizures by the activation of thalamocortical pathways likely occurred through a cortical feedforward inhibitory mechanism. Thalamocortical feedforward inhibition has been related to inhibitory GABAergic interneurons in the cortex [42, 43]. GABA_A_ receptor-mediated IPSCs were abolished by the GABA_A_ antagonist picrotoxin in thalamocortical slices [18]. However, whether GABA_B_ receptors mediate thalamocortical feedforward inhibition is still unclear. GABA receptors play an important role in mediating seizure activity. GABA_A_ receptors mediate 4-AP-induced synchronous events in the hippocampus and entorhinal cortex. GABA_A_ receptors also participate in the initiation, maintenance, and termination of ictal discharges [44, 45]. GABA_B_ receptors mediate 4-AP-induced long-lasting depolarization and asynchronous excitatory and inhibitory potentials in the rat hippocampal region [46]. CGP35348 blocks presynaptic GABA_B_ receptors and leads to an increase in GABA release and elevation of [K^+^]_o_ [47]. In the present study, GABAergic interneurons in the nRT were activated by blue light, which strongly hyperpolarized **V**B neurons, results in the generation of Ca^2+^ spikes, and triggers rebound burst firing in the somatosensory cortex. [48]. We hypothesize that 4-AP-induced seizures in the cortex are suppressed by cortical GABAergic neurons that receive inputs from the VB and release GABA. Ventrobasal inputs affect both cortical excitatory and inhibitory cells. Our results showed that the GABA_B_ antagonist CGP46381 had no effect on seizure activity when it was applied alone (Fig. 7b), thus excluding the possible confounding effects of cortical disinhibition. The involvement of GABA_B_ receptors in cortical feedforward inhibition has been reported previously, in which GABA_B_ but not GABA_A_ receptors regulate thalamocortical inputs of cortical neurons presynaptically and modulate the thalamocortical excitation of excitatory and inhibitory neurons in layer IV of the barrel cortex [49].

GABAergic interneurons and glutamatergic principal cells are excited by each other. Their interaction is reciprocal, and cortical interneuron excitation can directly suppress local networks through inhibitory feedback. Cortical interneurons can be excited by receiving long-range excitatory inputs from other subcortical nuclei, thus generating feedforward inhibition [50]. Thalamocortical axons were afferents on cortical fast-spiking inhibitory neurons but not afferent in regular-spiking neurons which are pyramidal neurons in rodents [51]. Thalamocortical relay neuron activation corresponds with synchronous discharges of S1 cortical neurons, leading GABAergic interneurons in the S1 region to release GABA and inhibit postsynaptic neurons [42]. Spiny fast-spiking interneurons receive thalamocortical neuron inputs and mediate IPSCs. Thalamocortical relay neurons can precisely and reliably activate spiny fast-spiking interneurons in the S1 region and provide feedforward inhibition of postexcitatory neurons [52]. Feedforward inhibition plays an important role in mature cortical function. GABA_A_ receptor-mediated transmission regulates thalamocortical circuit function. GABAergic transmission was also shown to be strongly engaged in stellate cells that received strong thalamocortical inputs in mature mice, whereas this engagement was weaker in neonatal mice [53]. These studies suggest that 4-AP-induced seizure activity was suppressed by the excitation of cortical interneurons through thalamocortical inputs and a mechanism of feedforward inhibition.

Our results showed that light stimulation affected thalamocortical relay neurons by activating ChR2-expressing neurons in the nRT. High-frequency and long-duration light stimulation may be more effective in suppressing cortical seizure-like activity. Histological examination verified that the stimulation of areas near the nRT and thalamus were more effective in suppressing cortical seizures, and GABA_B_ receptors may participate in suppressing seizure-like activity.

## Conclusions

The reticular thalamic nucleus (nRT) is a potential target for deep brain stimulation. It can affect ventrobasal thalamic regions, thus regulating the somatosensory cortex through the thalamocortical pathway. The present study found that evoking the activity of specifically nRT neurons by light stimulation of ChR2 protein attenuated cortical seizures. The mechanism of these suppressive effects involved γ-aminobutyric acid-B (GABA_B_) receptors in cortical regions. The findings suggest that neuronal firing in the nRT mediates cortical GABA neurons, which may be an effective target for deep brain stimulation to suppress cortical seizures.
